# Enabling interspecies epigenomic comparison with CEpBrowser

**DOI:** 10.1093/bioinformatics/btt114

**Published:** 2013-03-29

**Authors:** Xiaoyi Cao, Sheng Zhong

**Affiliations:** Department of Bioengineering, University of California San Diego, La Jolla, CA 92093, USA

## Abstract

**Summary:** We developed the Comparative Epigenome Browser (CEpBrowser) to allow the public to perform multi-species epigenomic analysis. The web-based CEpBrowser integrates, manages and visualizes sequencing-based epigenomic datasets. Five key features were developed to maximize the efficiency of interspecies epigenomic comparisons.

**Availability:** CEpBrowser is a web application implemented with PHP, MySQL, C and Apache. URL: http://www.cepbrowser.org/.

**Contact:** szhong@ucsd.edu

**Supplementary information:** Supplementary data are available at *Bioinformatics* online.

## 1 INTRODUCTION

Epigenomes play pivotal roles in cell identity, organismal development and disease processes (Bernstein *et al.*, 2007; Maunakea *et al.*, 2010), contribute to regulating cognition and behavior (Zhang and Meaney, 2010) and reflect personal variation (McDaniell *et al.*, 2010). By integrating environmental signals with genomic instructions, epigenomes are instrumental in bridging genotypic variation and phenotypic diversity. Leveraging a number of high-throughput sequencing enabled technologies, large international consortia are aiming to generate >1000 epigenomes from normal tissues and associated disease conditions (Abbott, 2011; Bernstein *et al.*, 2010; The ENCODE Project Consortium, 2004) [also see Box 1 in Satterlee *et al.* (2010)].

Analyzing and interpreting epigenomic information has become a pressing need. The successes obtained in associating epigenomic marks with genomic features inspired initial efforts of systematic annotation of chromatin states (Ernst and Kellis, 2010; Ernst *et al.*, 2011; Zhou *et al.*, 2011). However, our current capability of interpreting epigenomic information is still primitive. The concept of using cross-species comparison to annotate epigenomic functions may revolutionize epigenomic analysis (Feng *et al.*, 2010; Mikkelsen *et al.*, 2010; Milosavljevic, 2010; Xiao *et al.*, 2012). Indeed, by exploiting the link between evolutionary selection and regulatory functions, ‘comparative epigenomics’ have annotated epigenomes with unprecedented details (Xiao *et al.*, 2012).

We have developed the Comparative Epigenome Browser (CEpBrowser) to allow the public to perform multi-species epigenomic analysis. The web-based CEpBrowser extends the concepts of the UCSC and Ensembl Genome Browsers (Flicek *et al.*, 2011; Kent *et al.*, 2002; Zhu *et al.*, 2009) to integrate, manage and visualize large sequencing-based datasets for cross-species comparisons.

## 2 DESIGN PRINCIPLE AND KEY FEATURES

**Fig. 1.**
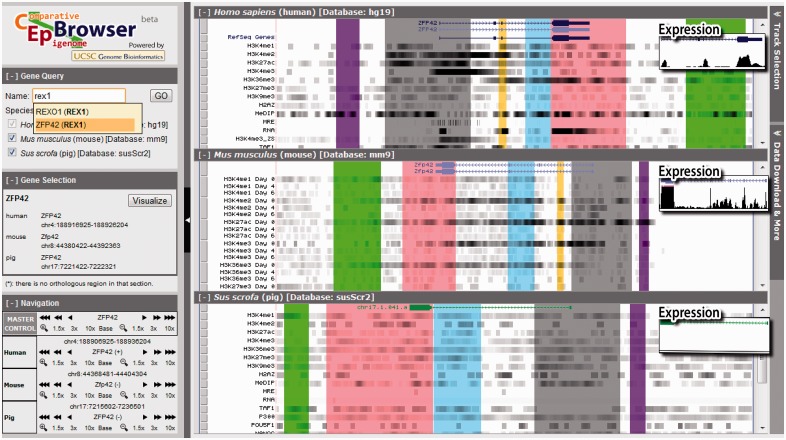
The multi-species alignment track. The main visualization area is split into three vertical panels, showing the genomes and epigenomes of humans, mice, and pigs. The top panel displays a human genomic region near the ZFP42 gene. Unfiltered sequencing data from ChIP-seq and other technologies are shown in epigenomic tracks (H3K4me1 etc.) in dense view in grayscale. The darkness reflects the number of overlapping sequencing reads at a genomic location. The middle and lower panels show the epigenomic data on comparable genomic regions in mice and pigs. Orthologous sequences are marked by the same color. These color-coded blocks clarify what epigenomic data are on orthologous sequences and thus comparable. On the left are three auxiliary panels for searching genes, selecting and navigating genomic regions, and controlling the synchronization of navigation (Master control). On the right is the button to call out the Track Selection Panel (hidden), which controls the track display and synchronization. The expression tracks show the RNA-seq data in the full view (inserts)

The design principle of CEpBrowser is to maximize the efficiency of interspecies epigenomic comparisons. Five key features were designed based on this principle. First, the epigenomic data from multiple species are presented side-by-side (Fig. 1, right). This simultaneous presentation of non-genome-sequence features of multiple species is different from other genome browsers (Kent *et al.*, 2002; Zhu *et al.*, 2009), which display one genome at a time as the basis, and they superimpose other information onto this basis. To present multi-species data in parallel, CEpBrowser vertically divides its visualization area into multiple panels, using each panel to display the genomic annotation and epigenomic features of one species. CEpBrowser allows user to specify any genomic region of interest, and it displays this region in a panel. In the meantime, CEpBrowser finds and visualizes comparable genomic regions in other species in the other panels. The comparable genomic regions are identified by an algorithm that maximizes the number of orthologous sequences (color blocks, Fig. 1) in the user’s region of interest. The epigenomic data of the other species are then superimposed onto these comparable genomic regions. The default presentation of the epigenomic data is a compact form [the ‘dense view’ (Kent *et al.*, 2002)], allowing for visually comparing as many epigenomic features across species as possible.

The second key feature is the automatic color-coding of epigenomic data based on the orthology of their underlying genomic sequences. The same color is used to shade orthologous sequences and the epigenomic data on the orthologous sequences in every species. This allows users to easily compare the epigenomes within each color block. For example, the strong signals of H3K4me2/3 and H3K27ac in the promoter of human ZFP42 (gray block, top, Fig. 1) are conserved as strong signals in mice (gray block, Day 0 tracks, middle panel), but they are not conserved in pigs (gray block, bottom). Gain or loss of orthologous sequences can also be easily spotted (yellow blocks).

The third key feature is the synchronized navigation of multiple species. When activated, this feature allows users to navigate the genome and epigenomes of a species while automatically tracing and visualizing the genomes and epigenomes of other species. For example, when the user zooms, shifts or jumps her view of the human genome and epigenomes, the views of mouse and pig genomes and epigenomes are updated in synchrony. This synchronization feature maximizes the number of orthologous sequences in the user’s view.

The fourth key feature is the synchronization of epigenomic tracks. Users can turn off the epigenomic tracks that are specific to some species, thus leaving the shared epigenomic tracks easily comparable. More importantly, the tracks of the same epigenomic mark can be simultaneously displayed or hid in every species. Finally, epigenomic tracks are automatically sorted into the same order in every species.

The fifth key feature is a simultaneous display of different tracks with different formats. The RNA-seq tracks are displayed in the ‘full view’ (Expression panels, Fig. 1), whereas other tracks are displayed in the dense view. This is particularly useful because the dense view is needed for juxtaposing many epigenomic marks in multiple species, whereas the RNA-seq data require the full view to provide a good sense of the expression levels. This feature allows associating interspecies epigenomic changes with the evolutionary changes of gene expression. For example, the conservation patterns of H3K4me2/3 and H3K27ac in human, mouse and pig pluripotent stem cells are in concordance with the conservation pattern of ZFP42 expression levels. H3K4me2/3 and H3K27ac showed a strong–strong–weak pattern on human, mouse and pig orthologous regions upstream to ZFP42 (gray blocks, Fig. 1), which correlated to the high–high–low expression pattern of ZFP42 in the three species (Expression panels, Fig. 1).

Besides these five key features, users can interactively interrogate the data using a set of auxiliary panels. The Gene Query Panel (upper left, Fig. 1) allows searching by gene names. CEpBrowser will recommend gene names by text similarity while the user is typing (Supplementary Fig. S1). The user input is compared with gene aliases from NCBI and Ensembl gene databases, and all matching or partially matching results will be listed. When genes are found by aliases, the alias will be marked in parentheses next to the canonical names. Users can select to visualize any of the listed genes with the Gene Selection Panel (Supplementary Fig. S2). When multiple species are displayed in parallel, the user can redirect the displayed region of any species with the Navigation Panel (lower left, Fig. 1). The ‘master control’ tools provide a synchronized control of all species, allowing users to zoom or shift the displayed regions in parallel (Supplementary Fig. S3). The Track Selection Panel (shown as a button on the upper right corner in Fig. 1 and Supplementary Fig. S4) allows displaying or hiding any tracks, or a group of related tracks. It also allows changing track display between full and dense formats (Supplementary Fig. S5).

## 3 DATA SUPPORT

CEpBrowser supports data download and data submission. Users can access the ‘data download and more’ panel to download all data tracks in CEpBrowser. Incorporation of new public data is enabled by an automated pipeline that converts mapped sequencing reads into the WIG format that can be managed and displayed on CEpBrowser. CEpBrowser has incorporated 70 ChIP-seq, RNA-seq, MRE-seq, MeDIP-seq datasets in three mammalian species (Supplementary Tables S1 and S2) (Chen *et al.*, 2008; Goren *et al.*, 2010; Lister *et al.*, 2009; Rada-Iglesias *et al.*, 2011; Xiao *et al.*, 2012; Yu *et al.*, 2013), allowing for comparing the epigenomes of pluripotent stem cells and different differentiation routes. Future work includes expanding to other species and other cell types. These developments require the community’s efforts in generating comparable epigenome maps in multiple species.

*Funding*: NSF DBI 09-60583.

*Conflict of Interest*: none declared.

## Supplementary Material

Supplementary Data
