# Supplementary material for: Enabling interspecies epigenomic comparison with CEpBrowser
Source: Bioinformatics. 2013 Mar 29;29(9):1223–5. doi: 10.1093/bioinformatics/btt114 (PMC3634190; doi:10.1093/bioinformatics/btt114)
Supplement: Supplementary Data [file supp_29_9_1223__index.html]

Enabling interspecies epigenomic comparison with CEpBrowser — Enabling interspecies epigenomic comparison with CEpBrowser — Supplementary Data 

# Enabling interspecies epigenomic comparison with CEpBrowser

## Supplementary Data

files

**Files in this Data Supplement:**

- Supplementary Data - pdf file
