# Supplementary material for: Enabling interspecies epigenomic comparison with CEpBrowser
Source: Bioinformatics. 2013 Mar 29;29(9):1223–5. doi: 10.1093/bioinformatics/btt114 (PMC3634190; doi:10.1093/bioinformatics/btt114)
Supplement: Supplementary Data [file supp_btt114_Supplementary-Figures-Text-2-5-final.pdf]

Supplementary Figures

Figure S1. Gene Query panel. The auto-completion feature of the Gene Query panel would search for partially matching gene names and aliases while the user is typing (A). The user can choose the species of interest for visualization (B).

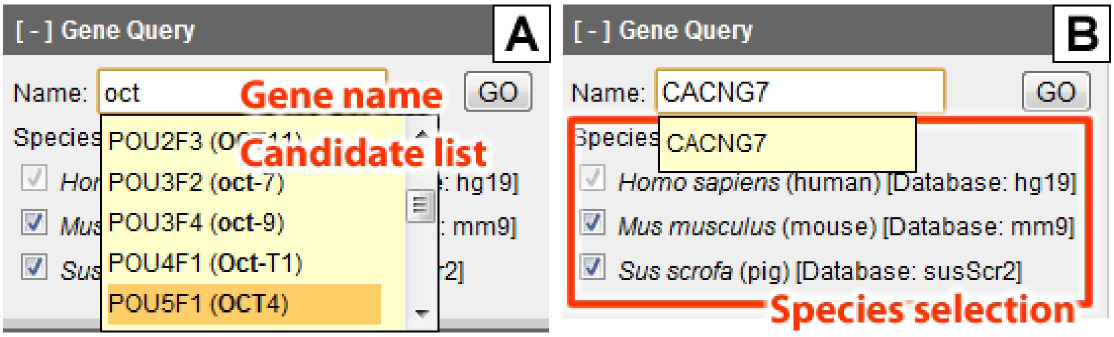

Figure S2. Gene Selection panel.

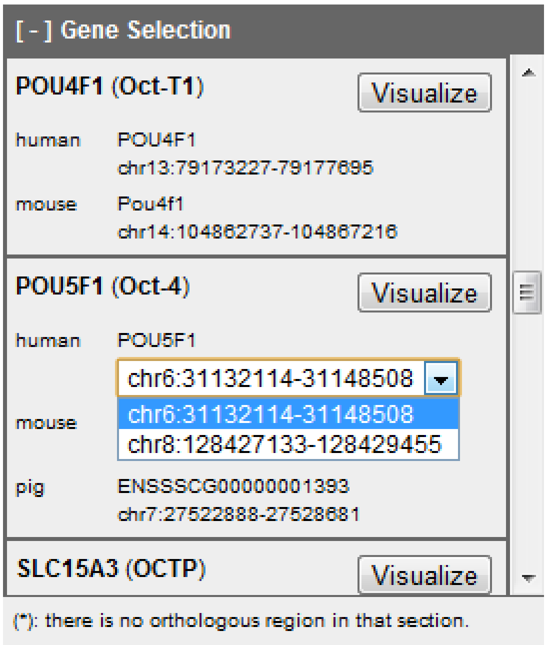

Figure S3. Navigation panel. Species-specific navigation buttons enable the user to shift, zoom, and jump the view to any genomic regions. The “Master control” enables synchronized navigation of the orthologous regions in multiple species. The POU5F1 gene is on the negative strands of the human and pig genomes (indicated by (-) next to the gene name). CEpBrowser automatically adjusts for strand differences and synchronizes the navigation to maximize the number of orthologous sequences in the view. When the user chooses a function in the master control (see the highlighted button), different actions are taken for each species (see the automatically highlighted buttons in concordance with the strands).

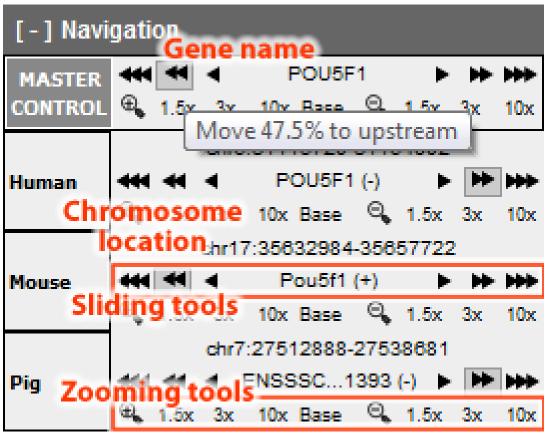

Figure S4. Track Settings panel. Users can show and hide tracks. This panel shows which epigenomic marks are shared by multiple species (data available in multiple species, Common tracks) and which epigenomic marks are specific to a species (Unique tracks).

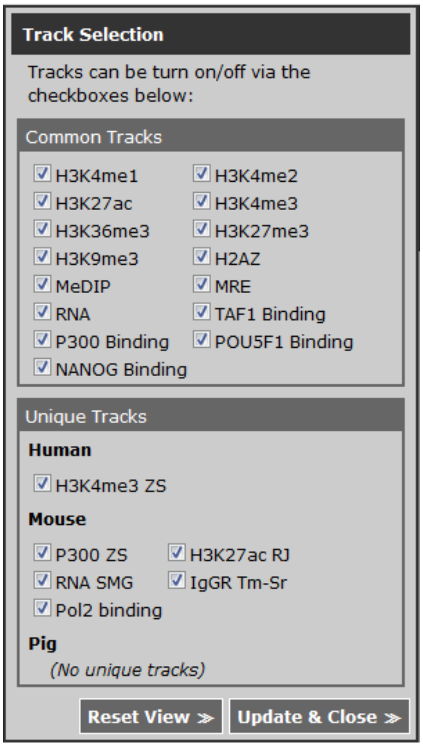

Figure S5. Full view and dense view. The time-course H3K4me2 data at 0, 4, and 6 days of differentiation of embryonic stem cells are displayed in full view (mountain shaped tracks). The height of these tracks represents the number of sequence reads (y-axis). The height is computed as $\ln(1+x)$, where x is the number of overlapping sequence reads. Pink lines mark places where the peaks are trimmed. Other epigenomic marks are displayed in dense view.

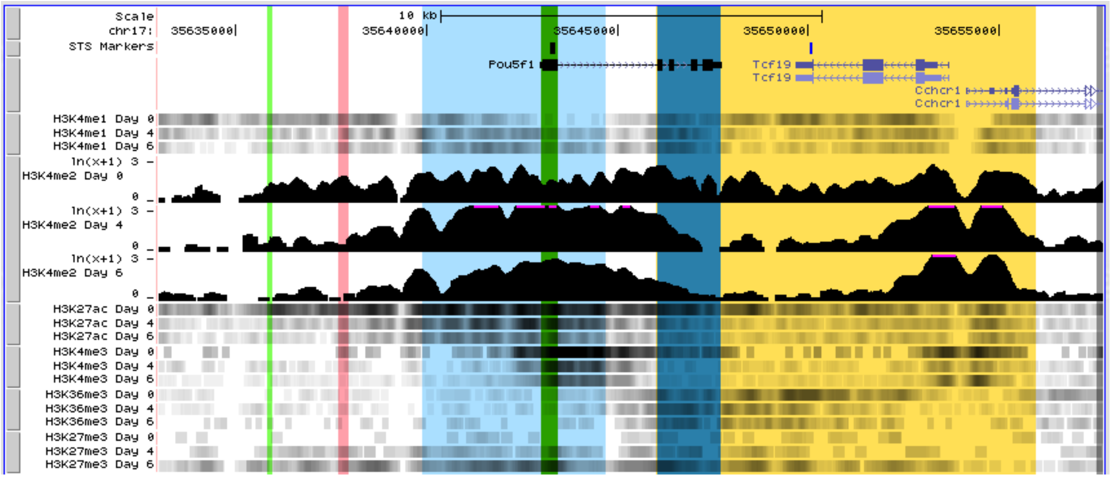

Figure S6. Color-coded multi-species alignments. (A) The main visualization area is vertically divided into panels. Each panel displays the data of one species. Species name and related information are shown on top of each panel (collapsible by clicking the name). At the bottom of each panel is a multi-species alignment track. This track shows the genomic segments that have orthologous sequences in other species. The same name and the same color are used to mark the orthologous sequences in every species. Clicking the blue buttons on the left of the epigenomic tracks will call out the Track Information & Settings Panel (Figure S7). (B) The color palette for marking orthologous sequences.

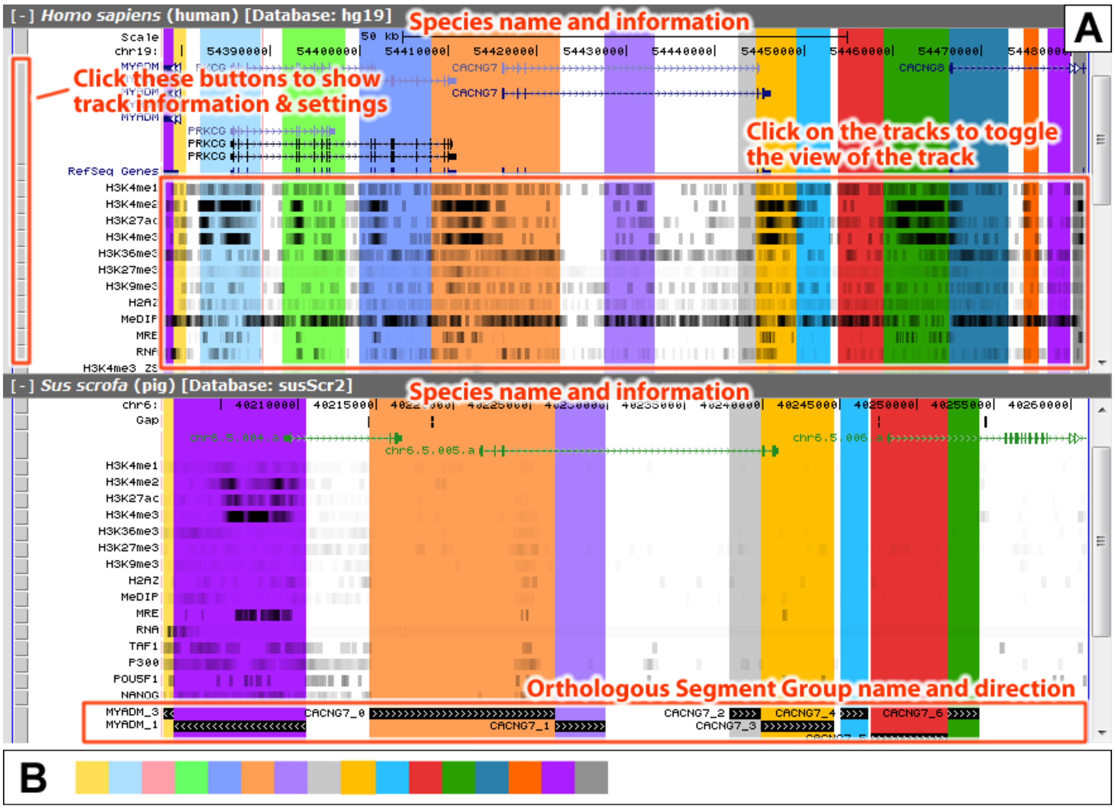

Figure S7. Track Information & Settings panel. This panel provides detailed information for the selected track. The settings of each track can be adjusted.

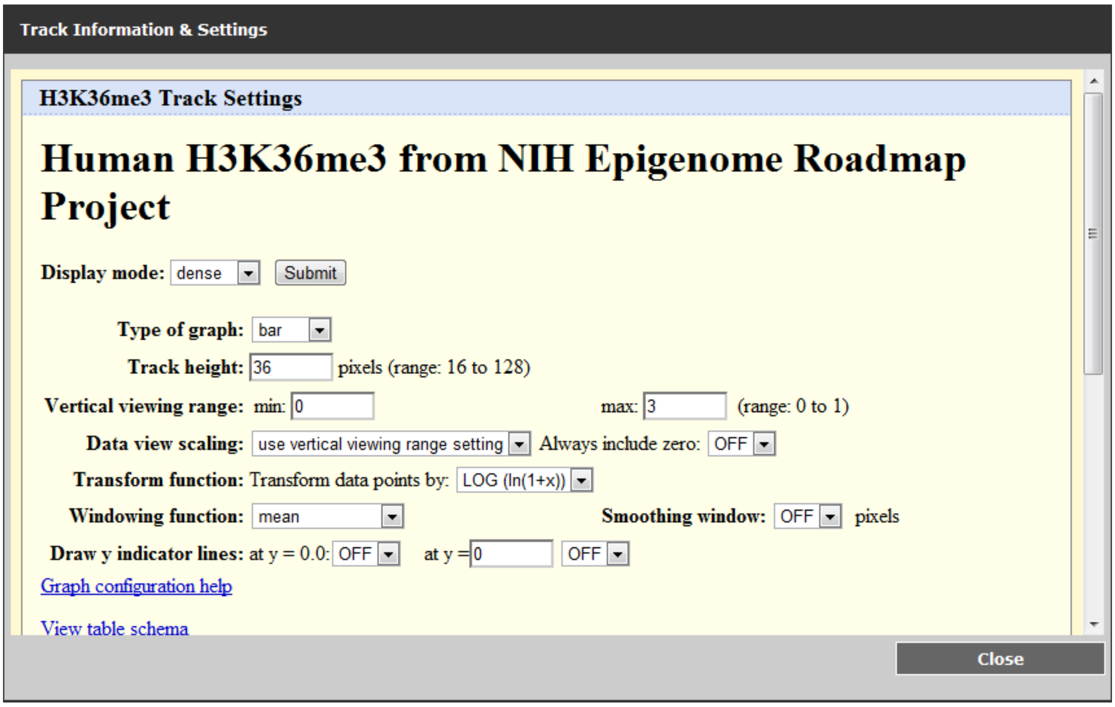

Figure S8. Generating the comparable genomic regions of three species. Orthologous sequence pairs were obtained from the chain files generated by UCSC liftOver program [1]. Each orthologous sequence pair is show in one color (left). The grey blocks represent sequence gaps between neighboring orthologous sequence pairs. Two rules were applied to generate three-species comparable genomic regions. The first is induction rule: any mouse segment and any pig segment would be determined as orthologous when according to the chain file they were both orthologous to the same human segment (green segments, right panel). The second is gap elimination. Small gaps (less than 1200bp) between neighboring orthologous sequence pairs were eliminated (marked by +, left panel). The arrow represents this merging process.

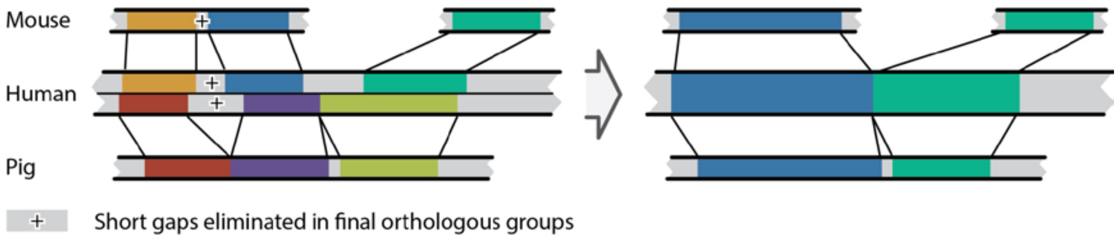

Supplementary Text

Data generation

An **orthologous gene group** contains a group of genes that are orthologous to each other. Orthologous gene pairs between any two of the species were retrieved from Ensembl Database (http://www.ensembl.org) [2] and then assembled into orthologous gene groups. In case of one-to-many and many-to-many orthologous mapping, CEpBrowser provides every gene in an orthologous gene group in the Gene Selection Panel and allows the user to choose which genes to visualize.

Aligned sequence groups are orthologous genomic sequences. Each aligned sequence group together with the epigenomic information on this group is colored with a distinct color. This color is used consistently in all species. The aligned sequence groups were determined as follows. First, pairwise alignable sequences between two species were retrieved from the UCSC Genome Browser chain files, which were computed by liftOver [1]. Second, these pairwise alignable sequences were merged into multi-species aligned sequence groups. When the gaps between two aligned sequence groups were shorter than 1200 bp in every species, the two groups were merged into one aligned sequence group (Figure S8). When a gene does not have aligned sequence groups in its 10,000 bp sequence neighborhood, this gene will be marked with an asterisk (*) in the Gene Selection Panel.

Available Datasets

CEpBrowser currently supports three species, which are Homo sapiens (human, reference genome GRCh37/hg19) [3], Mus musculus (mouse, reference genome NCBI37/mm9) [4], and Sus scrofa (domestic pig, reference genome SGSC Sscrofa9.2/susScr2) [5].

A total of 10 epigenomic data tracks, 5 transcription factor binding tracks and an RNA-seq track are shared in all three species (Table S1). Additional tracks were also built to accommodate species-specific data, including the data from the differentiation of human embryonic stem cells (hESC) to nasal epithelial cells (hNEC) [6], and the data from the differentiation of mouse [7] (Table S2).

Using CEpBrowser

CEpBrowser consists of five panels: gene query panel, gene selection panel, navigation panel, visualization panel, and two folded panels: track selection panel

and track information & settings panel. The left panels can be folded so that all the panels can be accessible under smaller screen resolution. The entire left panel group can also be hidden to provide larger space for the visualization panel.

Searching genes with the Gene Query Panel

A simple to start using CEpBrowser is to type a gene name in the Gene Query panel. CEpBrowser employs AJAX to show a gene name candidate list (see the yellow box in Figure S1A) when partial (at least 2 characters long) gene name is given.

The Species Selection boxes are used to select the species to be visualized in the browser. Species names and database versions are shown beside the boxes (Figure S1B). At least two species have to be checked before continuing.

Click GO to continue.

Viewing query results in the Gene Selection Panel

After a query is submitted, the selected orthologous gene group will be shown in the gene selection panel and will be shown in the visualization panel as well. If an incomplete gene name was provided as the query, all orthologous gene groups whose gene name or any one alias in human partially match the query (for example, "POU5F1" will match query "pou", "ou5f", “oct” for alias “oct4”, etc. The match is case-insensitive.) would be listed. The user should select which orthologous gene group to visualize.

The Gene Selection Panel shows the gene name in all selected species together with the chromosome locations for every matched orthologous gene group (Figure S2Figure S2). For genes without names in any species, the Ensembl IDs will be shown instead.

Due to incomplete genome annotation, sometime a gene is annotated with multiple genomic coordinates by Ensembl. When this happens, a drop-down menu for chromosomal locations will appear for the user to choose. The chromosomal locations with asterisks (*) indicate places where no aligned sequence groups were available.

The user can proceed by clicking the Visualization button.

Visualizing orthologous gene groups in the Visualization Panel

The genomes of all the selected species are shown in a side-by-side view. Each orthologous segment group is shaded in a distinct color. There are a total of 16 colors in CEpBrowser (Figure S6B). If the number of orthologous segment groups in the view is less than 16, every group is assigned with a unique color. When there are

more than 16 orthologous segment groups in the view, a track called "Multi-species Alignment Track" will appear in the bottom in each species' panel, showing the name and the direction of each orthologous segment group (Figure S6A).

CEpBrowser is built on the source code of UCSC Genome Browser [8] and virtually all operations of UCSC Genome Browser can be conducted in any of the species shown in the visualization panel, including zooming, moving coordinates, and ordering the tracks by drag-and-drop.

The Visualize button in the gene selection panel can be used to reset the current view.

To see the track information, the user can click the track name above its control of track visibility in the lower part of the page. The pop-up window will show data source, GEO accession number and references if any.

Navigating genomes with the Navigation Panel

The navigation panel provides control buttons to navigate genomes (Figure S3). The buttons in MASTER CONTROL trigger synchronized actions in each species. Other control buttons execute species-specific navigation functions. Sliding controls move the viewing regions to upstream or downstream regions. Zooming controls change the lengths of the genomic regions. When a sliding button in the MASTER CONTROL is pressed, the direction of move in each species is with respect to the direction of the gene in that species. For example, if a gene is on the Crick strand (as indicated by "-" in the navigation panel), sliding upstream in MASTER CONTROL will cause the view in this species to move to the right.

Controlling track displays with the Track Selection Panel

The track selection panel manages track visibility (Figure S4). This panel is hidden by default, and it can be called out by clicking the arrow sign on the upper right corner of the visualization panel.

All the data tracks available in CEpBrowser are listed in the track selection panel. By checking or unchecking the boxes next to each track, that track will be displayed or hidden in the visualization panel. Tracks are categorized into common tracks and species-specific tracks. When comparable experiments were carried in all species, the data from these experiments are listed as common tracks. One way to focus on interspecies comparison is to hide species-specific tracks.

The Reset View button can be used to reset default options, which will show all available tracks in their default order.

This panel will be automatically hidden after a user action is executed.

Detailed track adjustments with Track Information & Settings Panel

The track information & settings panel provides detailed information of each track and allows for delicate adjustments to the track display (Figure S7). This panel is hidden by default and can be called out by clicking the grey button on the left side of each track. This panel will be hidden with a user action or by clicking of the Close button.

References to supplementary text

1. Hinrichs, A.S., et al., *The UCSC Genome Browser Database: update 2006.* Nucleic acids research, 2005. **34**(suppl 1): p. D590-D598.
2. Flicek, P., et al., *Ensembl 2011.* Nucleic acids research, 2011. **39**(suppl 1): p. D800-D806.
3. International Human Genome Sequencing Consortium, *Initial sequencing and analysis of the human genome.* Nature, 2001. **409**(6822): p. 860-921.
4. Mouse Genome Sequencing Consortium, *Initial sequencing and comparative analysis of the mouse genome.* Nature, 2002. **420**(6915): p. 520-562.
5. UCSC Genome Bioinformatics Group. *UCSC Genome Browser Acknowledgments*. Available from: http://genome.ucsc.edu/goldenPath/credits.html#pig_credits.
6. Rada-Iglesias, A., et al., *A unique chromatin signature uncovers early developmental enhancers in humans.* Nature, 2011. **470**(7333): p. 279-283.
7. Xiao, S., et al., *Comparative Epigenomic Annotation of Regulatory DNA.* Cell, 2012. **149**(6): p. 1381-1392.
8. Kent, W.J., et al., *The human genome browser at UCSC.* Genome Res, 2002. **12**(6): p. 996-1006.
